# Book Reviews

**Published:** 1823-01

**Authors:** 


### MEDICAL AND SCIENTIFIC BIBLIOGRAPHY.

Vexat censura . . . . . . Corvos.

Art. I.—*Pharmacologia; comprehending the Art of Prescribing upon fixed and scientific Principles; together with the History of Medicinal Substances.* By J. A. Paris, M.D. F.R.S. &c. &c. 2 vols. Fifth Edition, enlarged.—8vo. pp. 870. London: Phillips, and T. and G. Underwood. 1822.

A work that has reached the fifth edition in so short a space of time as the Pharmacologia, does not stand in need of any commendation from us: nor is it within the reach of our censure, even if we thought it deserving of any. The public have already decided upon its merit; and we have but the humble task left us of announcing the additions and improvements which it has received, and to show the profession our high opinion of its value, by recommending it as promptly as possible to their notice. It will be observed, that the work has now grown into two volumes; the fourth edition comprising 578 pages only, the present one occupying 870, exclusive of a very copious index. The last edition, it appears, had been out of print nearly a twelvemonth; and Dr. Paris accounts for the delay in the appearance of the present one, "from an anxious desire to render the work worthy of the very liberal patronage which it has received from the medical profession." The increased size of the work, he tells us in his advertisement, proceeds, "in some measure, from the communications he has received from English practitioners, and from the recently-extended researches of the French and German chemists; but principally from the further development of those views respecting the *primary* and *secondary* operations of medicinal bodies, which were announced, but not explained, in the former editions." In comparing the two editions, we do not find, in the introduction, many alterations to notice: there are, however, a few omissions and some additions. The first striking improvement which occurs to us, immediately follows the introduction, and consists of an extended dissertation, comprising upwards of 120 pages, "on the operations of medicinal bodies, and on the classifications founded on them." Some important remarks on the *modus operandi* of medicinal agents bring us to the three principal arrangements of the materia medica,—viz. Cullen's, Dr. Young's, and that of Dr. Murray, whose plan is preferred by our author, "for its simplicity," and strict conformity to the views he intends to offer. The following short quotation will show the general outline of this classification:

"Dr. Murray observes that, in this arrangement, he places in the first division those substances which exert a GENERAL STIMULANT operation on the system. Of which there are two subdivisions, the Diffusible and the Permanent; the former including the class of Narcotics, with which may be associated, as not very remote in their operation, the class of Anti-spasmodics; the latter comprising two classes, viz. Tonics and Astringents. Through these there is a gradual transition, from the most highly diffusible stimulant to those most slow and durable in their action.

"A second division comprehends LOCAL STIMULANTS; those, the action of which is determined to particular parts of the system. Such are the classes of Emetics, Cathartics, Emmenagogues, Diuretics, Diaphoretics, Expectorants, and Sialogogues; with which may be associated the classes of Errhines and of Epispastics, founded on direct local application.

"The remaining classes include substances which do not operate according to laws peculiar to the living system. To one division may be referred those whose effects depend on the CHEMICAL changes they produce in the fluids or solids; the classes which may be established on this principle are Refrigerants, Antacids, Lithontriptics, and Escharotics. To another division belong those, the operation of which is purely MECHANICAL, as Anthelmintics, Demulcents, Diluents, Emollients, and certain Laxatives."

The division on the Theory and Art of Prescribing is also in many parts much extended, and, towards the conclusion, enriched by remarks upon remedies of external application, including Collyria, Lotions, Cataplasmata, &c.

The collection of Prescriptions is augmented by the addition of nearly fifty formulæ, arranged according to the plan sketched by Dr. Murray, and which concludes the first volume.

The second volume consists entirely of an alphabetical and detailed account of the different substances comprised in the materia medica. The principal additions are a long and interesting article on the Hydrocyanic Acid, an extension of that on Arsenic, an account of all the more modern preparations of the Salts of Cinchona; some few additional remarks on the Extractum Conii, on the Oxymuriate of Mercury, on the Liquor Calcis, on the Oleum Amygdalæ Amaræ Volatile; an additional article on the Oleum Tiglii (Croton); besides numerous notes in illustration of the subjects discussed, and a very extended account of the principal nostrums vended in this country.

We have said enough, we hope, to induce our readers to become better acquainted with this standard production, which should find a place in every medical library. A longer account would be superfluous, as we cannot pretend to give a detailed abstract of a work, every page of which is replete with interesting and valuable matter.

---

ART. II.—*Pharmacopœia Imperialis, sive Pharmacopœiæ Londinensis, Edinburgensis, et Dublinensis, collatæ, &c.*—pp. 255. Londini, 1823.

THIS little volume, which would at any time have been a valuable present to those who, from inclination or other motives, are anxious to become acquainted with pharmacy, is particularly well-timed at present, when the London Pharmacopœia is very difficult to be procured. We have occasion to know that many young men, who have the prospect of being subjected to an examination at Apothecaries' Hall, in which they are expected to be quite at home on all points of pharmacy, have been put to serious inconvenience from the cause above mentioned. Fortunately, the "Pharmacopœia Imperialis" relieves them from this difficulty, and we can confidently recommend it to them as possessing great advantages. It consists of the formulæ of the London, Edinburgh, and Dublin Colleges; the first being printed in a larger type, by which it is easily distinguished. The Materia Medica contains the synonyms and English names of the different articles; while the chemical changes which take place in the "præparata et composita" are explained in English, in clear and concise terms, giving altogether great facilities to the young pharmacologist, for whom it is intended, and to whom we again recommend it.

---

ART. III.—*A System of Osteology, comprising the Bones and Articulations of the Human Skeleton; to which is added, a Description of the Organs of Sight and Hearing, and the Peculiarities of the Fœtus. The whole intended and arranged as an Appendix to the "London Dissector," to supply its Deficiencies, and render it a complete Compendium of Anatomy.*—pp. 99. London: Burgess and Hill. 1822.

IT is obvious that a work of the above description cannot be the subject of analysis, and scarcely of criticism. If the descriptions be accurate, and the language perspicuous and plain, every thing is done that can be reasonably expected; and we are of opinion that the little volume before us answers fairly to this description. In it the usual course pursued in elementary works is reversed, as the Osteology is now published as the conclusion of a compendium of Anatomy; but it must be recollected that this work was written to assist *the Dissector,* who meets with the descriptions in the order that he would examine the various component parts of the body, and therefore that order is substantially correct, and completes the author's design.

We have lately heard, with equal surprise and regret, that subjects for dissection have become extremely scarce in this metropolis, and that most enormous prices are now demanded and obtained. We lament this circumstance, as injurious to the profession especially, as well as to the best interests of humanity,—although this latter expression may, to some unprofessional persons, appear somewhat paradoxical. We have heard, on very good authority, that fourteen guineas have been lately demanded for a subject; and we cannot help suggesting the advantage that would accrue to those who are anxious to live, if the bodies of those unfortunate persons who destroy themselves could be made available for the purposes of anatomical instruction. The very prospect of such a destination might, in some instances, prevent the commission of the rash act; and thus the result of such a regulation would, in either case, be beneficial to society.

The Bibliographical Notice of Dr. VENABLES' Lecture on *Oxalic Acid,* is unavoidably deferred for want of room, notwithstanding the *two additional half-sheets* given in this Number.]

